# Educational needs and training conditions of young clinical neurophysiologists: survey of IFCN-young neurophysiologists network

**DOI:** 10.1016/j.cnp.2026.01.009

**Published:** 2026-02-01

**Authors:** Orsolya Györfi, Thananan Thammongkolchai, Ricardo Kienitz, Nortina Shahrizaila, Hatice Tankisi, Anita Kamondi

**Affiliations:** a Neurology Unit, Department of Neurosurgery and Neurointervention, Semmelweis University, Amerikai út 57., 1145 Budapest, Hungary; b Neurology Division, Department of Medicine, Ramathibodi Hospital, Mahidol University, 270 Rama VI Rd, Thung Phaya Thai, Ratchathewi, Bangkok 10400, Thailand; c Department of Neurology, University Hospital, Goethe University, Germany Haus 95, Schleusenweg 2-16, 60528 Frankfurt am Main, Germany; d Neurology Unit, Department of Medicine, Faculty of Medicine, University of Malaya, 50603 Kuala Lumpur, Malaysia; e Department of Clinical Medicine, Aarhus University, Palle Juul-Jensens Boulevard 99, 8200 Aarhus N, Denmark; f Department of Clinical Neurophysiology, Aarhus University Hospital, Palle Juul-Jensens Boulevard 99, 8200 Aarhus N, Denmark; g Department of Neurology, Semmelweis University, Balassa u. 6., 1083 Budapest, Hungary

**Keywords:** Clinical neurophysiology, Specialty training, Education, IFCN-YNN, Survey

## Abstract

**Objectives:**

In 2022, the International Federation of Clinical Neurophysiology (IFCN) launched the Young Neurophysiologists Network to support the representation of its younger members. This survey assessed the training of early-career clinical neurophysiologists and identify unmet needs.

**Methods:**

A 31-item online questionnaire was distributed to IFCN members under 40 years of age, evaluating educational goals, mentorship, expertise levels, and preferences for e-learning.

**Results:**

From 57 countries 331 participants responded. Marked regional differences emerged in training quality, confidence, and needs. In high-income countries, satisfaction with core modules—EEG, ENG–EMG, and video-EEG—was highest, while lower ratings were reported in low-income countries, especially for EEG. Training priorities also varied: neuromuscular ultrasound was the most requested topic in both high- and middle-income countries, while low-income countries prioritized video-EEG and evoked potentials. Mentorship significantly improved continued training. Online education was used by 58%, with 64% preferring IFCN Masterclasses.

**Conclusion:**

Young neurophysiologists show strong interest in advanced training, particularly in neuromuscular ultrasound. Expanding mentorship and access to IFCN resources could help address global disparities.

**Significance:**

This survey highlights significant regional differences in young neurophysiologists’ training and underscores the need to expand mentorship and equitable access to advanced educational resources.

## Highlights

• Economic status determines the training priorities in clinical neurophysiology.
• Mentorship supports the continued training of young clinical neurophysiologists.
• Among IFCN educational resources, online Masterclasses are the most frequently used.

## 1 Introduction

The International Federation of Clinical Neurophysiology (IFCN) established the Young Neurophysiologist Network (YNN) in 2022 to create a global network for young clinical neurophysiologists. YNN aims to advocate for their interests within IFCN activities and enhance networking opportunities for these young clinicians.

The IFCN actively promotes educational initiatives in various formats, offering recommendations for harmonization standards for specialized training in clinical neurophysiology (CN) among member societies. According to these proposals, the training should begin with achieving basic competence in the core modules, including electroencephalography (EEG), electroneurography and electromyography (ENG-EMG), and evoked potentials (EP), followed by gaining knowledge in complementary modules that require competence in associated primary modules (Cole and Kamondi, 2023).

The IFCN Europe, Middle East and Africa chapter (EMEAC), Asia-Oceania Chapter, and Latin America Chapter published a survey on education and training practices in clinical neurophysiology, covering a broad range of variations among member countries in the status of specialty and training, competency, accreditation, and practice (Cole et al., 2022).

Inspired by the diverse intercontinental educational contexts mentioned above, the YNN aimed to describe the current status and educational needs of young IFCN members (under 40 years of age) from various backgrounds.

## 2 Methods

The survey was created by the 2022–2024 YNN Officers (O.G., T.T., R.K.) with support from senior IFCN members (A.K., H.T., N.S.), and consisted of 31 questions (Appendix 1).

We collected demographic information, including age (stratified into three groups: ≤29 years, 30–34 years, and 35–39 years), gender, and details about the participants’ current workplace.

The participants were asked to provide information regarding:

− the current state of their training program (number of specialty examinations, current training status, and whether they have a mentor).

− the number of yearly performed examinations.

− their self-perceived confidence and comfort in technical skills (perceived expertise level and need for additional supervision in primary and complementary CN modules).

− training satisfaction (expertise in subfields, expectations about the specialty training program).

− e-learning preferences.

− migration plans.

Likert scales (1 = very well to 5 = not at all) were used to assess how well the training program prepared participants for complex neurophysiological tasks and fulfilled their educational expectations across different fields.

For data collection, we distributed an online survey via email to all registered members of the International Federation of Clinical Neurophysiology (IFCN), inviting young members (≤40 years) to complete the 31-question questionnaire hosted on SurveyMonkey. The invitation included a brief description of the study and a direct survey link. The first question of the survey asked for the participant’s age, ensuring only respondents aged 40 years or less were included.

The survey invitation reached approximately 20,000 registered IFCN members, of whom 338 completed the survey. Seven respondents were aged 40 or older and were excluded from the analysis. Because IFCN does not maintain individual member age data, the exact response rate could not be calculated.

The survey was not limited to YNN members but was sent to all IFCN-registered individuals to ensure broad international representation. Although IFCN is a federation of societies, all registered members had individual email accounts through which the survey was distributed.

Data were analysed using GraphPad Prism software. The primary analysis focused on describing the current status, educational needs, and learning opportunities among an international group of young clinical neurophysiologists.

The primary analysis of the current paper focuses on the existing status, educational needs, and opportunities of an international group of young clinical neurophysiologists.

## 3 Results

### 3.1 1. Demographic characteristics

**Fig. 1:**
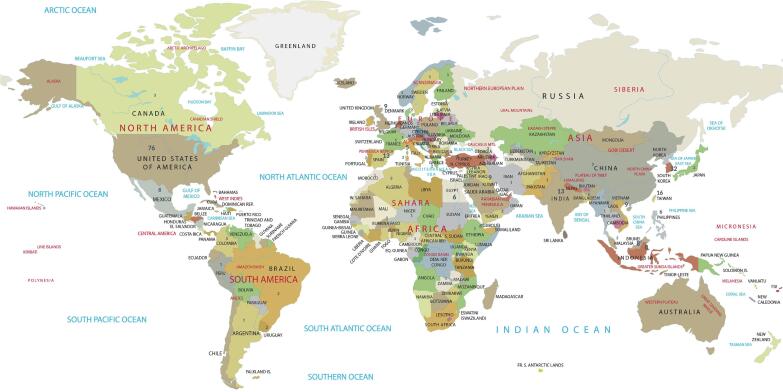

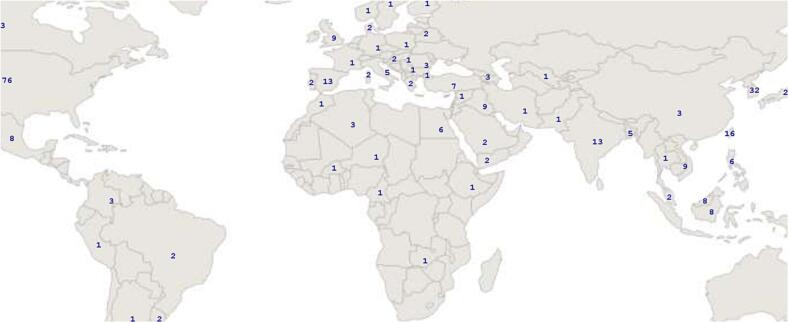

A total of 331 participants under the age of 40 from 57 countries completed the survey. Of these, 263 (79 %) identified their original continental regions. The largest group of participants (42 %) was from Asia, followed by those from the Middle East (Fig. 1).

The majority of the participants belonged to the age group 30 to 34 years (152 participants, 46 %; 56 % female, 44 % male), followed by those aged 35 to 39 years (145 participants, 44 %; 58 % female, 42 % male). The youngest group, consisting of those aged 29 years or younger, made up 10 % (34 participants, 35 % female, 65 % male) of the participants.

Of the 279 participants who responded to the survey question on certification, 162 (58 %) reported being certified in Clinical Neurophysiology. Among these, 99 also held certification in neurology, 1 in neurosurgery, 5 in rehabilitation medicine, and 8 had a second certification that was not specified.

The age group analysis revealed that the highest percentage of participants certified in CN were those between 35 and 39 years old (49 %), followed by those aged 30 to 34 years (45 %). The youngest group (29 years or younger) had a certification rate of 6 %.

### 3.2 Continuing education

We examined the characteristics of individuals who reported continuing training after the CN specialty examination.

Across all age groups, the presence of a mentor was associated with a clearly higher likelihood of continued training. Among mentored participants, 40–53 % of those aged ≤ 34 years and 25 % of those aged 35–39 years were certified and still in training, compared with only 14–17 % in the same age groups without a mentor. Similarly, mentored colleagues more frequently reported ongoing training while non-certified (18–30 %) than those without mentorship (7–25 %). In contrast, participants without mentors more often reported being non-certified and no longer in training (33–47 %), whereas this proportion dropped to 4–12 % among those with mentors. (Table 1.).

**Table 1:** Data show the distribution of participants by age group and mentorship status, indicating continuation of clinical neurophysiology (CN) training. Mentorship is associated with a higher proportion of participants continuing training after certification across all age groups.

Mentorship status / Age group	Certified, still training (%)	Certified, no more training (%)	Non-certified, training (%)	Non-certified, no training (%)
No mentor				
≤29 years (n = 2)	50	–	–	50
30–34 years (n = 24)	17	25	25	33
35–39 years (n = 28)	14	32	7	47
With mentor				
≤29 years (n = 20)	40	20	30	10
30–34 years (n = 66)	53	17	18	12
35–39 years (n = 59)	25	42	24	4

Analysis of data on the influence of mentors on being in training (despite already acquired CN speciality certificate) across continents confirms that having a mentor enhances learning aspirations in both certified and non-certified colleagues (Table 1).

Our data shows that the presence of a dedicated mentor is more likely in a tertiary referral center than in a non-referral hospital (76 % vs. 56 %).

### 3.3 Expertise level in primary and complementary CN modules

**Fig. 2:**
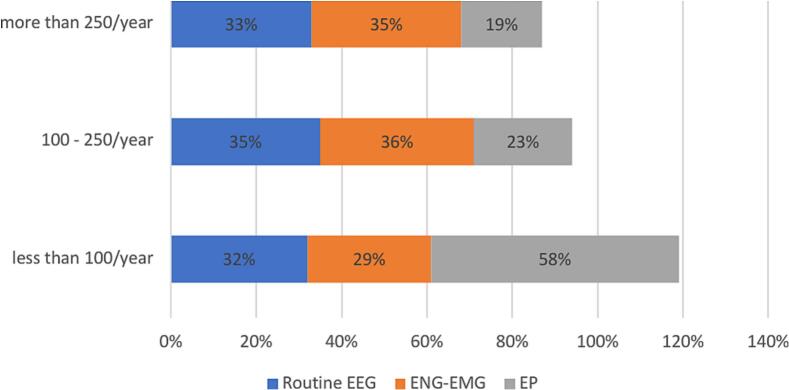

**Fig. 3:**
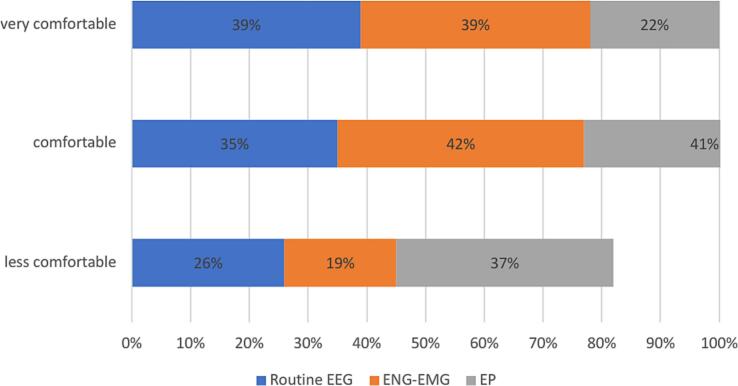

We evaluated the number of examinations performed by the responders in one year (Fig. 2) and self-percived confidence level in primary CN modules (Fig. 3).

Experience and confidence levels varied across modalities. ENG-EMG and EEG had the highest exam numbers (>250 exams: 35 % and 33 %, respectively), while EP was less common (only 19 %). Most participants had limited exposure to EP, with 58 % performing fewer than 100 exams. Confidence mirrored experience: 39 % reported high-confidence with EEG and ENG-EMG, but only 22 % with EP. A higher proportion reported low confidence with EP (37 %) compared to EEG (26 %) and ENG-EMG (19 %). These findings highlight limited training and confidence in EP relative to EEG and ENG-EMG.

We examined the association between confidence levels and the number of yearly examinations performed in primary modules.

Confidence in EEG skills increases with the number of examinations. Only 17 % of those performing < 100 EEGs/year reported high confidence, rising to 40 % with 100–250 EEGs and 60 % with > 250. Low confidence decreases from 47 % to 13 % as experience grows (Table 1).

Similarly, confidence in performing ENG-EMG exams increases with experience. Among participants conducting fewer than 100 exams per year, 42 % reported low confidence, while only 25 % felt highly confident. This changes significantly in the 100–250 range, where just 11 % reported low confidence and 30 % felt highly confident. Among those performing over 250 exams annually, 62 % reported high confidence, and only 7 % expressed low confidence.

Likewise, confidence in EP exams increases with experience: 52 % of those performing.

<100/year reported low confidence, while only 11 % felt highly confident. For > 250/year, 51 % reported high confidence and just 14 % low confidence (Table 2) (Supplementary file 1).

**Table 2:** Confidence in function of yearly performed examination in primary modules.

	Less than 100/year			100–250/year			More than 250/year		
	EEG	ENG-EMG	EP	EEG	ENG-EMG	EP	EEG	ENG-EMG	EP
Less confident	47 %	42 %	52 %	17 %	11 %	21 %	13 %	7 %	14 %
Confident	36 %	33 %	37 %	43 %	59 %	53 %	27 %	31 %	35 %
Very confident	17 %	25 %	11 %	40 %	30 %	26 %	60 %	62 %	51 %

**Fig. 4:**
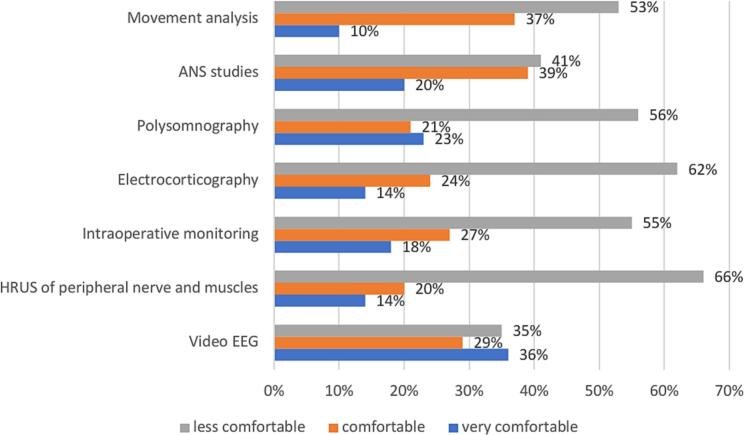

Assessing the expertise level in complementary CN modules among 179 responders (54 % of the total), video-EEG analysis was identified as the technique with highest confidence (36 %), while high-resolution ultrasonography of peripheral nerves and muscles (HRUS) was noted most often as the area of lowest confidence (66 %) (Fig. 4).

### 3.4 Fulfilment in CN training

We analyzed the training satisfaction in primary and complementary CN modules and aimed to determine whether differences in satisfaction could be affected by the economic status of participant's countries of origin. To do this, we focused on the subset of respondents who provided both training feedback and country information, which allowed us to evaluate data from 164 participants (50 % of the total sample). For the analysis, countries were categorized according to the World Bank's income classification: high-income, low-income, and a combined middle-income group (including both upper-middle and lower-middle income economies) (https://blogs.worldbank.org/en/opendata/world-bank-country-classifications- by-income-level-for-2024–2025).

**Fig. 5:**
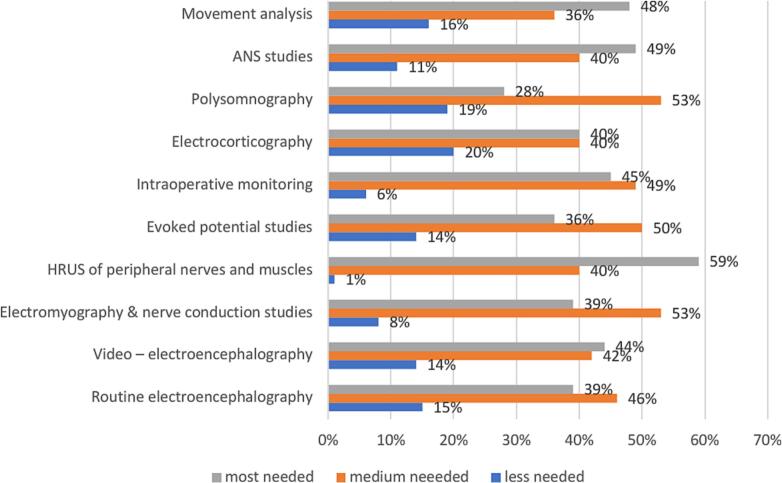

In high-income countries, HRUS of peripheral nerves and muscles appears to be the most desired area for training (59 %), followed by autonomic nervous system (ANS) studies (49 %) and movement analysis (48 %). (Fig. 5).

**Fig. 6:**
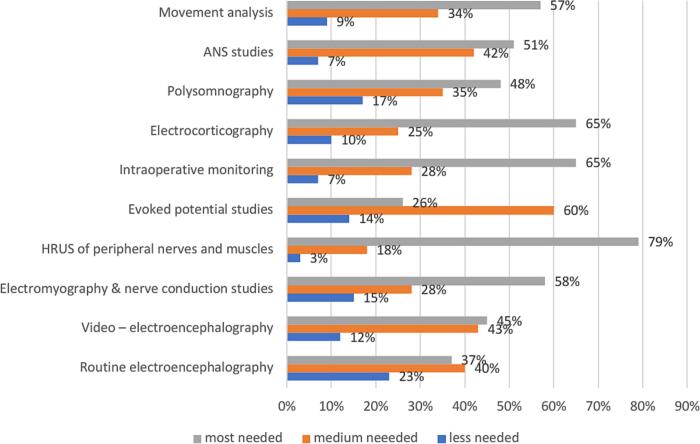

Similarly, in middle-income countries, the most desired training area is HRUS of peripheral nerves and muscles, selected by 79 % of responders, followed by equally highly prioritized intraoperative monitoring and electrocorticography (65 %) (Fig. 6).

**Fig. 7:**
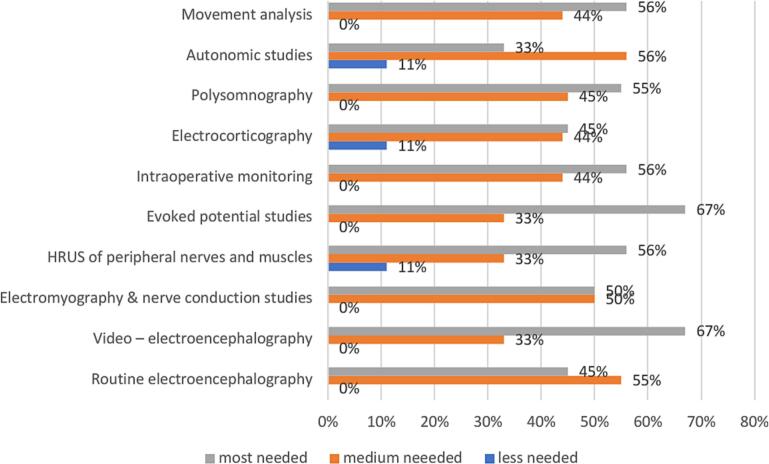

In low-income countries, the most frequently identified training need is video −EEG and EP studies (67 %), followed by intraoperative monitoring, movement analysis and HRUS of peripheral nerves and muscles (56 %) (Fig. 7).

We analyzed responses from 164 participants who reported both their educational needs and country of origin, allowing us to assess satisfaction with training across different income-level regions. Across all income groups, the basic training modules consistently received the highest satisfaction ratings.

**Fig. 8:**
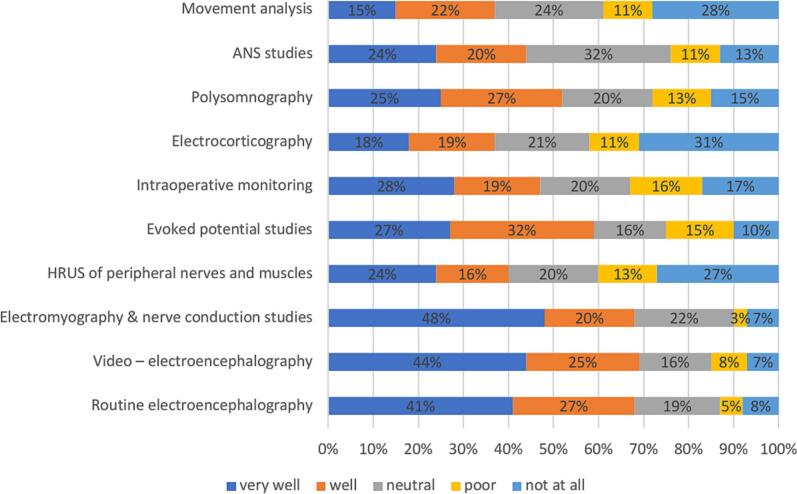

In high-income countries, about 70 % of participants rated parts of the basic modules— routine EEG and ENG-EMG, as well as video −EEG training—as “very well” or “well” (Fig. 8).

**Fig. 9:**
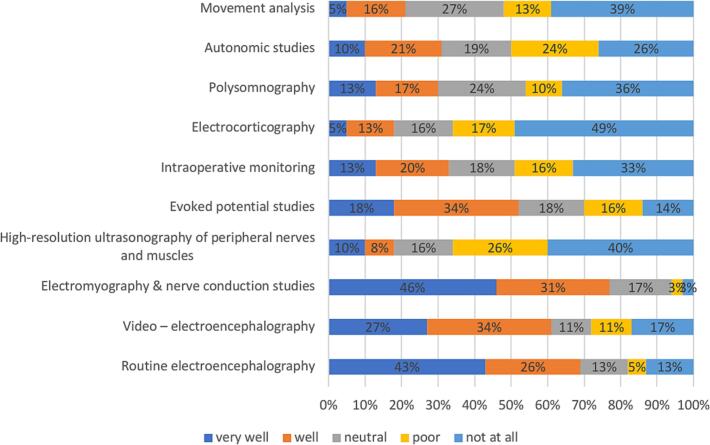

In middle-income countries, a similarly high satisfaction level was observed, but only for the basic modules (EEG and ENG-EMG) (Fig. 9).

**Fig. 10:**
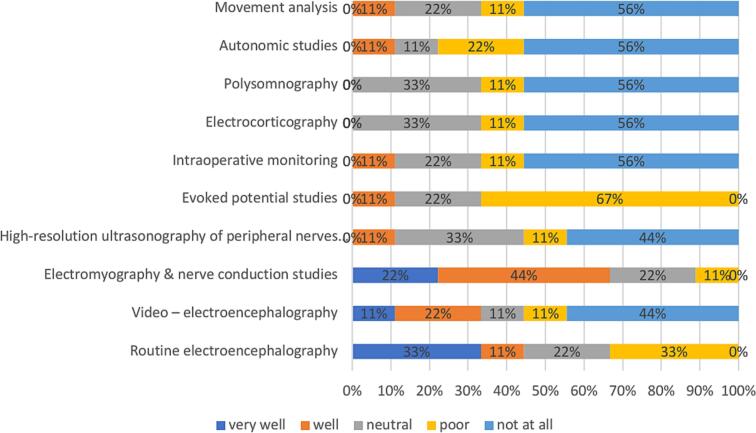

In low-income countries, ENG-EMG training received the highest satisfaction rating, with 68 % of participants marking it as “very well” or “well”, followed by EEG training at 44 % (Fig. 10).

We examined the progressivity levels of the workplaces of those responders who were the most satisfied (rated’very well’ and’well’) in primary modules.

**Fig. 11:**
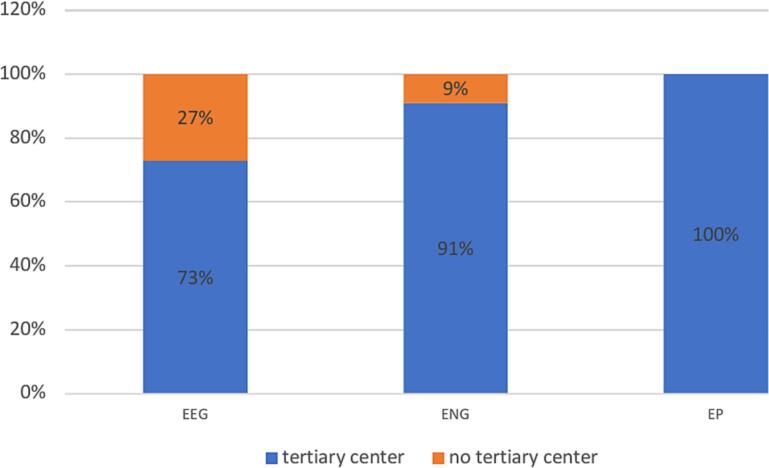

We found that a significant percentage of colleagues who are satisfied with the effectiveness of training (Likert scale’very well’ −’well’) are more likely to work in a tertiary hospital (Fig. 11.).

The results from a four-item Likert scale (very well, well, neutral, not at all) indicated that among 178 respondents, 42 % believed that the training program well prepared them to select and perform the most suitable CN techniques and to interpret their results in complex cases; of these participants, 71 % had a mentor.

### 3.5 E-learning preferences

Among the participants, 181 (58 %) responded to the questions related to online learning habits.

**Fig. 12:**
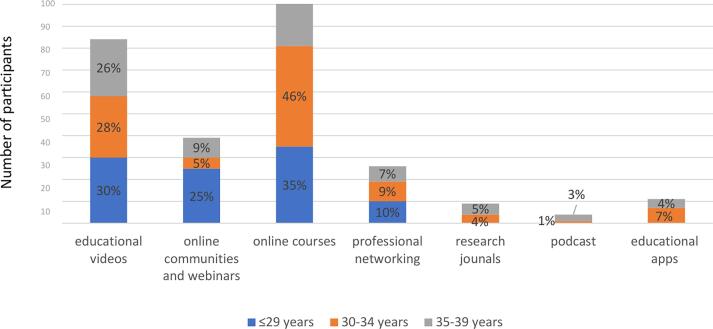

Analysis based on age groups revealed that individuals in the 30–34 age group are more inclined (47 %) to use online resources, with online courses (46 %) being the most favored option. In contrast, the youngest group (aged 29 and under) shows a preference for educational videos. Additionally, participation in professional networking as a resource of education declines with age (Fig. 12).

**Fig. 13:**
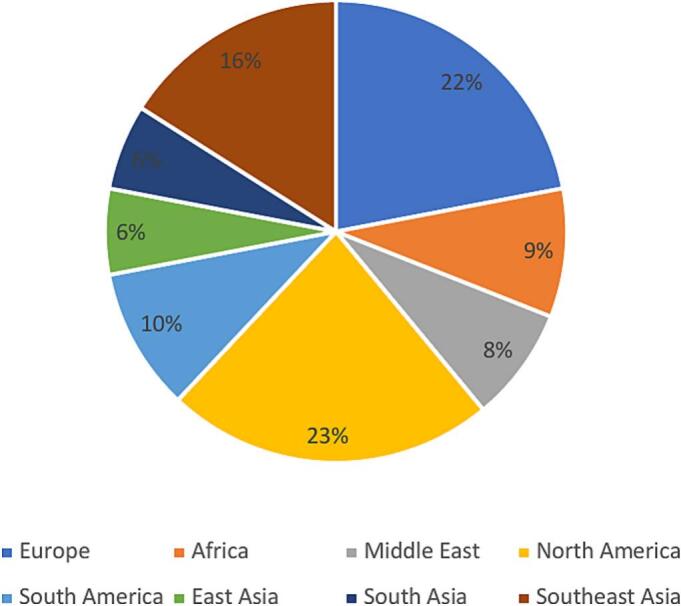

Of the 181 participants who responded to the online training habits survey, 121 (67 %) of the responders are aware of IFCN educational resources. Most users belong to the 30–34 and 35––39 age groups (45 % and 41 %, respectively), while awareness is less prevalent among the youngest (less than 29 years of age) (14 %). Among the users, 109 specified their geographical location; the majority (23 %) are from North America (Fig. 13).

**Fig. 14:**
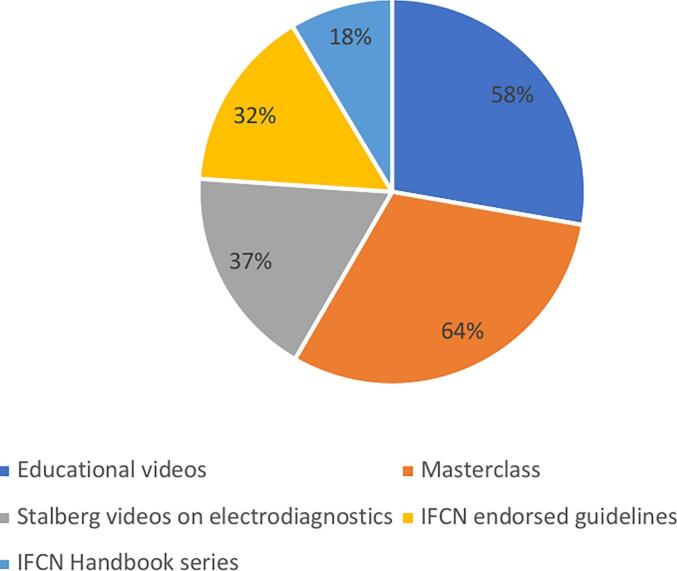

Among the IFCN educational resources, the Masterclasses are the most frequently used online educational materials (64 %) (Fig. 14).

## 4 Discussion

Our survey outlines the current status, training, and educational requirements of young clinical neurophysiologists at the international level.

In the first part of our survey, we reviewed the conditions and circumstances of participants who showed a strong interest in pursuing additional training after the CN specialty board exam.

The first influential factor is age: our results show that colleagues under 34 are more likely to continue training after the board exam, while the likelihood decreases significantly after 35 years of age.

The second influential factor is having a mentor. Mentorship greatly increases the young colleague’s enthusiasm for further training, regardless of their certification level. Our data across different continents confirms that mentorship boosts learning goals, especially in specialized centers where mentors are more readily available. For example, 71 % of those skilled in complex neurophysiology techniques had a mentor, showing a strong connection between mentorship and training quality.

Continuous education is crucial in medical training (Toh et al., 2022), particularly in neurophysiology, where staying up-to-date with knowledge and skills is essential (Samano et al., 2018). While various guidelines and educational review papers are available (Tatum et al., 2022, Hari et al., 2018, Peltola et al., 2023, Tankisi et al., 2019, Stalberg et al, 2019), hands-on experience and precision, such as in ENG-EMG and HRUS of peripheral nerves and muscles, can be acquired through mentorship, which helps to improve practical skills (Recker et al., 2024).

Recognizing this, the IFCN–EMEAC launched a Mentorship Program in 2023. This program provides young professionals with career guidance, networking opportunities, and support for career growth, ultimately building self-confidence and independence.

The program currently connects 22 mentors and 22 mentees from various European and Middle Eastern countries, including Denmark, Egypt, Finland, France, Georgia, Germany, Greece, Iraq, Italy, Nigeria, Norway, Poland, Portugal, Serbia, Scotland, Spain, Switzerland, Turkey, and the United Kingdom. This diverse group is actively involved in professional development.

The IFCN-EMEAC Mentorship program could offer professional guidance to colleagues in hospitals without referral options and limited access to specialists, enabling them to confidently adopt new examination techniques in clinical practice.

Our analysis of training satisfaction across primary and complementary CN modules revealed distinct patterns related to the economic status of respondent’s countries. High- and middle- income groups showed high demand for HRUS of peripheral nerves and muscles, which had the lowest competence levels and the greatest training demand. Recognized as a new tool in neurophysiology (Fionda et al., 2021), HRUS may become a core module alongside ENG-EMG examinations (Cole and Kamondi, 2023).

Low-income countries prioritize video-EEG training. Differences in resources and healthcare priorities explain why high-income countries focus on HRUS (Duncan and Rae, 2024) while low-income countries emphasize video-EEG as a cost-effective tool for key neurological diagnoses (Yadav et al, 2021).

Satisfaction with training in basic modules was generally higher across all income groups compared to the complementary modules, although the level of satisfaction was significantly greater among respondents working in tertiary hospitals.

Our results suggest that performing over 250 examinations annually in primary CN modules is linked to greater confidence in the field. EMEAC countries support high-level training (1,000 for EEG, 750 for ENG-EMG, and 500 for EP studies), acknowledging that these benchmarks may be difficult for some countries. The availability of international training centers and the development of personal training schemes through mentorship programs could offer a solution for this challenge (Cole at al., 2022, Minta et al., 2023).

When analyzing training satisfaction more closely, the young neurophysiologists pointed out that they were’not at all’ satisfied with the training in some complementary modules.

Electrocorticography, movement analysis, and HRUS of peripheral nerves and muscles were the three areas where colleagues felt the training was least satisfactory.

Recognizing the improbability that every trainee will reach the same level of competence in complementary fields, it would be optimal to receive basic training in complementary modules during CN training through focused hands-on workshops and supervised clinical practice (Cole and Kamondi, 2023).

A practical method for training involves e-learning options, which provide flexible approaches (Dong et al., 2021). Among our respondents, the 30–34 age group is most engaged in online learning, and online courses are their preferred choice. More than half of the responders used the extensive range of reliable teaching materials offered by the IFCN on its website; however, awareness of these educational resources remains quite low among the youngest generation.

This study offers valuable insights into the current status, training, and educational needs of young clinical neurophysiologists worldwide. However, some limitations must be acknowledged.

Firstly, the primary limitation stems from the sample's composition. As most respondents are from IFCN member societies, the findings may be biased towards individuals who already have access to basic and advanced neurophysiology programs. This self-selected sample might not fully represent the diverse experiences and challenges faced by young clinical neurophysiologists in regions or institutions not affiliated with IFCN.

Secondly, the research depends on self-reported data, which can be influenced by recall bias or social desirability bias. Although efforts were made to gather comprehensive data, the subjective nature of self-evaluation must be considered when analyzing the results, especially regarding training satisfaction and confidence levels.

Thirdly, because survey participation was open, some data points were not enough for analysis. For example, migration plans could not be properly evaluated since too few participants responded to that section to produce statistically meaningful results.

## 5 Conclusions

The study recognizes disparities in resources and priorities among countries with different income levels. While it highlights differences in training requirements (e.g., HRUS of peripheral nerves and muscles in high-income versus video-EEG in low-income countries), the survey may not fully capture the complex socioeconomic and infrastructural barriers that influence training access and quality across all regions. Further studies focusing on these details are needed.

Future research should further explore how international mentorship initiatives and standardized training pathways might help reduce the regional disparities identified in this survey. In particular, longitudinal studies could provide valuable insight into how early and sustained access to high-quality training influences long-term professional development, career retention, and clinical competence.

In parallel, the increasing role of digital education warrants closer examination. Future work should assess how online learning environments, including IFCN Masterclasses and virtual mentorship formats, can be better tailored to the needs of early-career neurophysiologists in different regions. Identifying barriers to access, participation, and sustained engagement will be crucial for the development of more inclusive and effective global training strategies.

## Funding sources

This research did not receive any specific grant from funding agencies in the public, commercial, or not-for-profit sectors. We declare that we have no competing interests in relation to this article.

## Declaration of Competing Interest

The authors declare that they have no known competing financial interests or personal relationships that could have appeared to influence the work reported in this paper.

## Glossary

ANS: autonomic nervous system CN: clinical neurophysiology EEG: electroencephalography
EMEAC: Europe, Middle East and Africa Chapter
ENG-EMG: electroneurography-electromyography
EP: evoked potentials
HRUS: high-resolution ultrasound
IFCN: International Federation of Clinical Neurophysiology YNN: Young Neurophysiologists Network

## Appendix A Supplementary data

Supplementary Fig. 1

Supplementary data1

## References

[b0005] Cole J., Kamondi A. (2023). A proposal for harmonizing clinical neurophysiology training in the Europe, Middle East and Africa chapter of the International Federation of Clinical Neurophysiology. Clin. Neurophysiol..

[b0010] Cole J., Kamondi A., Kimaid P.T., Shahrizaila N. (2022). Training and education practice in the Europe, Middle East and Africa, Latin America and Asia Oceania chapters, IFCN; an international survey. Clin. Neurophysiol. Pract..

[b0015] Dong L., Gao T., Zheng W., Zeng K., Wu X. (2021). E‐Learning for Continuing Medical Education of Neurology residents. Mind Brain Educ..

[b0020] Duncan N.W., Rae C.L. (2024). Geographical and economic influences on neuroimaging modality choice. R. Soc. Open Sci..

[b0025] Fionda L., Di Pasquale A., Morino S., Leonardi L., Vanoli F., Loreti S., Garibaldi M., Lauletta A., Alfieri G., Bucci E., Salvetti M. (2021). Changes of clinical, neurophysiological and nerve ultrasound characteristics in CIDP over time: a 3-year follow-up. J. Neurol..

[b0030] Hari R., Baillet S., Barnes G., Burgess R., Forss N., Gross J., Hämäläinen M., Jensen O., Kakigi R., Mauguière F., Nakasato N. (2018). IFCN-endorsed practical guidelines for clinical magnetoencephalography (MEG). Clin. Neurophysiol..

[b0035] Minta K.J., Sescu D., Da Luz D., Kaliaperumal C. (2023). Global Mentorship in Neurosurgery for Medical students Study (the GloMNMS Study): a multinational multi-institutional cross- sectional audit. BMJ Open.

[b0040] Peltola M.E., Leitinger M., Halford J.J., Vinayan K.P., Kobayashi K., Pressler R.M., Mindruta I., Mayor L.C., Lauronen L., Beniczky S. (2023). X Routine and sleep EEG: minimum recording standards of the International Federation of Clinical Neurophysiology and the International League against Epilepsy. Clin. Neurophysiol..

[b0045] Recker F., Neubauer R., Dong Y., Gschmack A.M., Jenssen C., Möller K., Blaivas M., Ignacio P.M., Lucius C., Ruppert J., Sänger S.L. (2024). Exploring the dynamics of ultrasound training in medical education: current trends, debates, and approaches to didactics and hands-on learning. BMC Med. Educ..

[b0060] Tankisi H., Pugdahl K., Beniczky S., Andersen H., Fuglsang-Frederiksen A. (2019). Evidence-based recommendations for examination and diagnostic strategies of polyneuropathy electrodiagnosis. Clin. Neurophysiol. Pract..

[b0065] Tatum W.O., Mani J., Jin K., Halford J.J., Gloss D., Fahoum F., Maillard L., Mothersill I., Beniczky S. (2022). Minimum standards for inpatient long-term video-EEG monitoring: a clinical practice guideline of the international league against epilepsy and international federation of clinical neurophysiology. Clin. Neurophysiol..

[b0070] Toh R.Q.E., Koh K.K., Lua J.K., Wong R.S.M., Quah E.L.Y., Panda A., Ho C.Y., Lim N.A., Ong Y.T., Chua K.Z.Y., Ng V.W.W. (2022). The role of mentoring supervision coaching teaching and instruction on professional identity formation: a systematic scoping review. BMC Med. Educ..

[b0080] Yadav H., Shah D., Sayed S., Horton S., Schroeder L.F. (2021). Availability of essential diagnostics in ten low-income and middle-income countries: results from national health facility surveys. Lancet Glob. Health.

