# Supplementary figures and images for: Educational needs and training conditions of young clinical neurophysiologists: survey of IFCN-young neurophysiologists network
Source: Clin Neurophysiol Pract. 2026 Feb 1;11:93–102. doi: 10.1016/j.cnp.2026.01.009 (PMC12907009; doi:10.1016/j.cnp.2026.01.009)

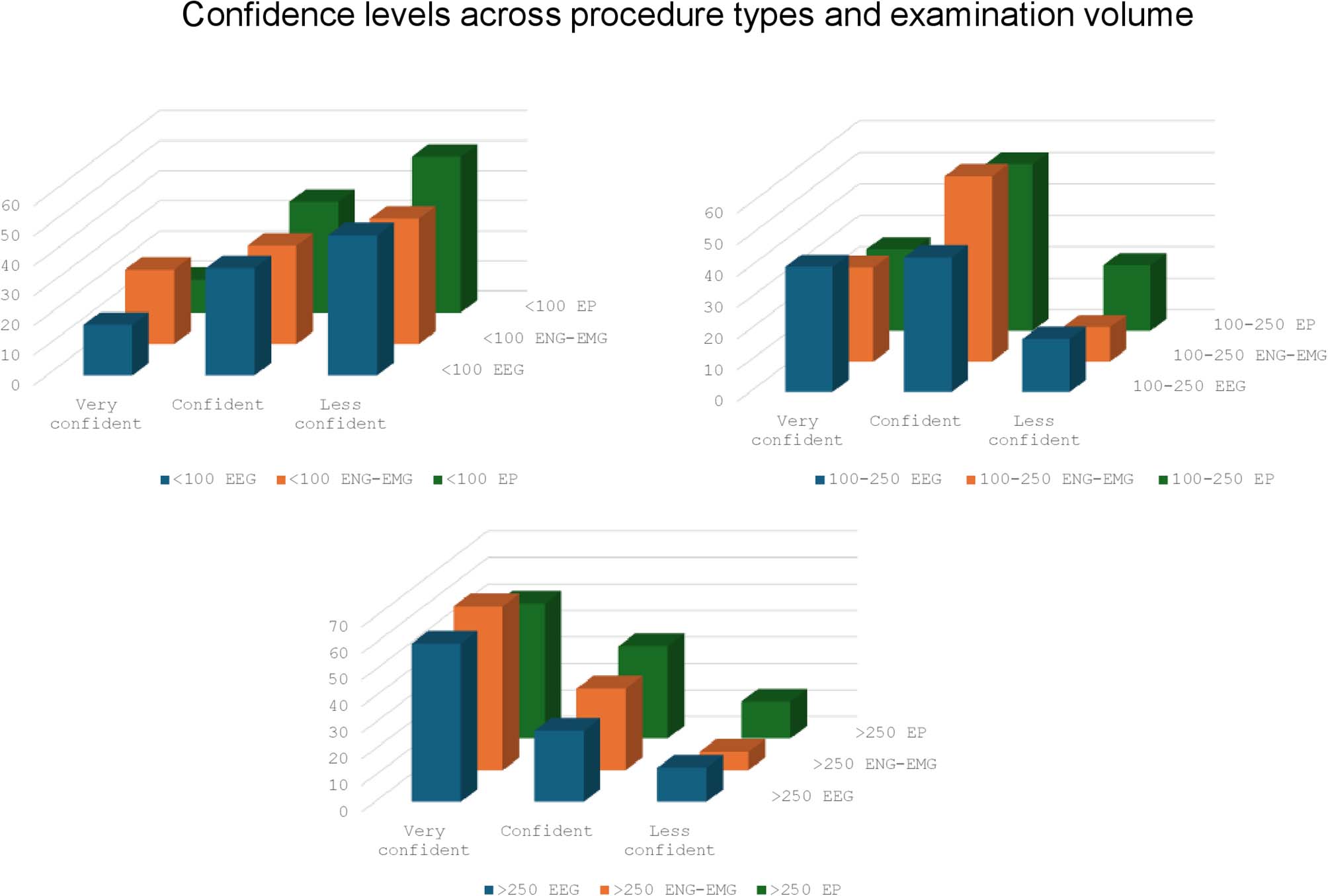

Supplement: Supplementary Fig. 1 [file mmc1.jpg]
