# Supplementary material for: Educational needs and training conditions of young clinical neurophysiologists: survey of IFCN-young neurophysiologists network
Source: Clin Neurophysiol Pract. 2026 Feb 1;11:93–102. doi: 10.1016/j.cnp.2026.01.009 (PMC12907009; doi:10.1016/j.cnp.2026.01.009)
Supplement: Supplementary data1 [file mmc2.doc]

**Appendix 1.**

YNN survey questions

Educational needs and training conditions of young clinical neurophysiologists - a survey of the International Federation of Clinical Neurophysiology-Young Neurophysiologists Network

Our aim is to survey the young neurophysiologists about their views on different topics, pertaining to their current working conditions, confidence in technical skills, e-learning preferences, migration patterns

**Socio-demographic characteristics**

1. Age

a.≤29

b. 30–34

c. 35–39

d. 40 and above

2. Sex

a. Male

b. Female

c: other

d: I do not wish to disclose

3. In which country are you currently working / affiliated?:

4. Are you still in clinical training?

a. Yes

b. No

5. If you are still in clinical training, for how long have you already been in training?

6. What is the total amount of expected / required training in Clinical Neurophysiology in your country?

7. How many other trainees of your level work at your centre?

8. Do you have a dedicated mentor responsible for your training?

a: Yes

b: No

9. Is Clinical Neurophysiology an independent speciality in your country

a.Yes

b. No

10. Do you have board certification in Clinical Neurophysiology?

a. Yes

b. No, but I have the intention to pursue

11: Do you have another board certification?

a: Yes

b: No

12: If so, in which additional specialty are you board-certified?

13. Do you work at a tertiary referral centre of Clinical Neurophysiology?

a. Yes

b. No

14: If Clinical Neurophysiology is no independent specialty in your country, do you work at a tertiary referral centre of Neurology?

a: Yes

b: No

**Self confidence in technical skills**

15. Please specify how many examinations do you perform in primary neurophysiological modules (1.:less than 100/year, 2.:100 - 250/year, 3.: more than 250/year)

a. Routine electroencephalography: ....

b. Electromyography & nerve conduction studies: ...

c. Evoked potentials: ……

16. Please specify your expertise level in these modules (1.: very comfortable, 2: comfortable, 3: less comfortable)

a. Routine electroencephalography: ....

b. Electromyography & nerve conduction studies: ...

c. Evoked potentials: ……...

17. Are you being trained or have you been trained in the therapeutic use of neurophysiological methods (e.g. therapeutic TMS, tDCS)

A: yes

B: no

18. If so, for how many months?

19. Please specify your expertise level in complementary modules (1.: very comfortable, 2: comfortable, 3: less comfortable)

a. Video - electroencephalography

b. High-resolution ultrasonography of peripheral nerves and muscles

c. Intraoperative monitoring

d. Electrocorticography

e. Polysomnography

f. Autonomic studies

g. Movement analysis

20. Do you think that in several neurophysiological fields further training/more supervision is needed (1. :most needed, 2.: medium needed, 3.: less needed)

a. Routine electroencephalography

b. Video - electroencephalography

c. Electromyography & nerve conduction studies

d. High-resolution ultrasonography of peripheral nerves and muscles

e.Evoked potentials

f. Intraoperative monitoring

g. Electrocorticography

h. Polysomnography

i. Autonomic studies

j. Movement analysis

21. How well did the training programme in Clinical Neurophysiology fulfil your educational expectation in different fields (1. very well, 2. well, 3. neutral, 4. poor 5. not at all)

a. Routine electroencephalography

b. Video - electroencephalography

c. Electromyography & nerve conduction studies

d. High-resolution ultrasonography of peripheral nerves and muscles

e. Evoked potentials

f. Intraoperative monitoring

g. Electrocorticography

h. Polysomnography

i. Autonomic studies

j. Movement analysis

22. How well did your training program prepare you to select, perform and interpret the results of the most suitable neurophysiological technique in a complicated case:

1. very well

2. well

3. neutral

4. not at all

**E-learning preferences**

23. What training material do you prefer as e-learning resource:

a. Online courses

b. Educational videos

c. Podcasts

d. Professional networking

e. Research Journals

f. Educational apps

g. Online communities and webinars

h: other:

24. What new training materials do you wish for?

25. Are you aware of IFCN online educational resources?

a. Yes

b. No

26. If yes which online IFCN resource do you prefer

a. Educational videos

b. Masterclass

c. Stalberg Videos on electrodiagnosis

d. IFCN Endorsed guidelines

e. IFCN Handbook series

**Migration pattern**

27. Intention to migrate abroad as a neurophysiologist:

a. Yes

b. No

28. The purpose to migrate:

a. For further specialty training

b. To work/practice as a neurophysiologist without further specialty training

29. When do you intend to migrate abroad

a. Within next 1 year

b. 1–2 years

c. 3–5 years

d. 6–10 years

30. The preferred destination to migrate:

a. Within your current country

b. USA

c. Australia

d. Canada

e. Within EU (please specify)

f. Other: (please specify)
